# Supplementary material for: Identification of Potential Probiotics Producing Bacteriocins Active against Listeria monocytogenes by a Combination of Screening Tools
Source: Int J Mol Sci. 2021 Aug 10;22(16):8615. doi: 10.3390/ijms22168615 (PMC8395247; doi:10.3390/ijms22168615)
Supplement: Supplementary file 1 [file ijms-22-08615-s001.zip › ijms-1336617-supplementary.pdf]

Supplementary data

Identification of lactic acid bacteria isolated from raw cows' milk producing bacteriocins active against *Listeria monocytogenes* using a combination of screening tools

Christian Desiderato[1], Steffen Sachsenmaier[1], Kirill V. Ovchinnikov[2], Jonas Stohr[1], Susanne Jacksch[3], Dominique N. Desef[1], Peter Crauwels, Markus Egert[3], Dzung B. Diep[2], Oliver Goldbeck[1], and Christian U. Riedel[1,*]

[1] Institute of Microbiology and Biotechnology, University of Ulm, Ulm, Germany
[2] Faculty of Chemistry, Biotechnology and Food Science, Norwegian University of Life Sciences, Ås, Norway
[3] Faculty of Medical and Life Sciences, Institute of Precision Medicine, Furtwangen University, Villingen-Schwenningen, Germany
* Correspondence: christian.riedel@uni-ulm.de; Tel.: +49-731-5024853

Supplementary Table S1: Identification of bacteria isolated from raw cow milk by 16S rDNA sequencing and MALDI-Biotyping.

Isolate	16S rRNA gene sequencing		MALDI-Biotyping		Final identification
	Closest database match	Identity (%)	Closest database match	score	
M1	*Pediococcus lolli*	100.00	-	1.26	*Pediococcus acidilactici*
M2	*Lactococcus lactis*	99.93	*Lactococcus lactis*	2.10	*Lactococcus lactis*
M3	*Pediococcus lolli*	100.00	*Pediococcus sp.*	2.27	*Pediococcus acidilactici*
M4	*Enterococcus faecium*	99.85	*Enterococcus faecium*	2.16	*Enterococcus faecium*
M5	*Enterococcus faecalis*	100.00	*Enterococcus faecalis*	2.13	*Enterococcus faecalis*
M6	*Lactococcus garvieae*	100.00	*Lactococcus garvieae*	1.89	*Lactococcus garvieae*
M7	*Lactococcus lactis*	99.78	*Lactococcus lactis*	2.38	*Lactococcus lactis*
M8	*Lactococcus garvieae*	100.00	*Lactococcus garvieae*	2.09	*Lactococcus garvieae*
M9	*Enterococcus faecium*	99.02	*Enterococcus faecium*	2.36	*Enterococcus faecium*
1	*Lactococcus lactis*	99.82	*Lactococcus lactis*	2.25	*Lactococcus lactis*
2	*Leuconostoc citreum*	99.79	*Leuconostoc citreum*	2.07	*Leuconostoc citreum*
3	*Leuconostoc citreum*	100.00	-	1.63	*Leuconostoc citreum*
5	*Lactococcus lactis*	100.00	*Lactococcus lactis*	2.31	*Lactococcus lactis*
6	*Lactococcus garvieae*	99.20	*Lactococcus garvieae*	1.92	*Lactococcus garvieae*
7	*Lactococcus garvieae*	99.93	*Leuconostoc citreum*	1.88	*Leuconostoc citreum*
9	*Lactococcus garvieae*	99.76	*Lactococcus garvieae*	1.73	*Lactococcus garvieae*
10	*Weissella paramesenteroides*	99.93	*Lactococcus garvieae*	1.80	*Weissella paramesenteroides*
11	*Lactococcus garvieae*	100.00	*Lactococcus garvieae*	2.00	*Lactococcus garvieae*
12	*Lactococcus garvieae*	99.64	*Lactococcus garvieae*	2.04	*Lactococcus garvieae*
13	*Pediococcus lolli*	99.85	-	1.59	*Pediococcus acidilactici*
14	*Corynebacterium glutamicum*	99.52	*Corynebacterium glutamicum*	1.87	*Corynebacterium glutamicum*
15	*Enterococcus italicus*	100.00	*Enterococcus italicus*	1.78	*Enterococcus italicus*
16	*Corynebacterium glutamicum*	99.83	-	1.35	*Corynebacterium glutamicum*
17	*Lactococcus garvieae*	99.93	-	1.45	*Lactococcus garvieae*
18	*Lactococcus lactis*	99.72	*Lactococcus lactis*	2.08	*Lactococcus lactis*

Table S1: Continued.

Isolate	16S rRNA gene sequencing		MALDI-Biotyping		Final identification
	**Closest database match**	**Identity (%)**	**Closest database match**	**score**	
19	*Leuconostoc citreum*	100.00	-	1.36	*Leuconostoc citreum*
20	*Lactococcus lactis*	97.57	-	1.32	*Lactococcus lactis*
21	*Lactococcus lactis*	100.00	-	0.00	*Lactococcus lactis*
22	*Lactococcus lactis*	98,83	*Weissella paramesenteroides*	2.07	*Lactococcus lactis*
23	*Enterococcus faecalis*	97.85	*Enterococcus faecalis*	2.31	*Enterococcus faecalis*
24	*Lactococcus lactis*	100.00	*Lactococcus garvieae*	1.83	*Lactococcus garvieae*
25	*Lactococcus lactis*	100.00	*Lactococcus lactis*	2.20	*Lactococcus lactis*
26	*Lactococcus garvieae*	99.93	-	1.48	*Lactococcus garvieae*
27	*Pediococcus lolli*	99.65	-	1.44	*Pediococcus acidilactici*
28	*Leuconostoc citreum*	100.00	*Leuconostoc citreum*	1.96	*Leuconostoc citreum*
29	*Weissella paramesenteroides*	99.93	-	1.35	*Weissella paramesenteroides*
30	*Leuconostoc citreum*	100.00	*Leuconostoc citreum*	1.79	*Leuconostoc citreum*
32	*Enterococcus faecalis*	99.81	*Enterococcus faecalis*	2.30	*Enterococcus faecalis*
33	*Weissella paramesenteroides*	99.42	-	1.66	*Weissella paramesenteroides*
34	*Enterococcus faecalis*	99.62	*Lactococcus garvieae*	1.86	*Enterococcus faecalis*
35	*Lactococcus petauri*	99.82	*Lactococcus garvieae*	1.73	*Lactococcus garvieae*
36	*Lactococcus lactis*	99.82	*Lactococcus lactis*	2.26	*Lactococcus lactis*
37	*Lactococcus lactis*	100.00	*Lactococcus lactis*	2.08	*Lactococcus lactis*
38	*Lactococcus lactis*	99.34	*Lactococcus lactis*	2.19	*Lactococcus lactis*
39	*Enterococcus faecalis*	99.91	*Lactococcus garvieae*	1.72	*Enterococcus faecalis*
40	*Lactococcus garvieae*	99.70	*Lactococcus garvieae*	1.82	*Lactococcus garvieae*
41	*Lactococcus lactis*	99.85	*Lactococcus lactis*	2.34	*Lactococcus lactis*
50	*Lactococcus garvieae*	99.85	*Candida kefyr*	2.11	*Lactococcus garvieae*
51	*Leuconostoc citreum*	99.93	-	1.59	*Leuconostoc citreum*
52	*Weissella paramesenteroides*	99.71	*Weissella paramesenteroides*	2.06	*Weissella paramesenteroides*
53	*Weissella paramesenteroides*	99.93	*Weissella paramesenteroides*	1.81	*Weissella paramesenteroides*
54	*Weissella paramesenteroides*	99.85	*Weissella paramesenteroides*	2.10	*Weissella paramesenteroides*

Table S1: Continued.

Isolate	16S rRNA gene sequencing		MALDI-Biotyping		Final identification
	**Closest database match**	**Identity (%)**	**Closest database match**	**score**	
55	*Weissella paramesenteroides*	99.93	*Weissella paramesenteroides*	2.02	*Weissella paramesenteroides*
56	*Lactococcus petauri*	99.16	*Candida kefyr*	2.37	*Lactococcus garvieae*
57	*Lactococcus lactis*	99.91	*Lactococcus lactis*	2.24	*Lactococcus lactis*
pos	*Pediococcus lolii*	99.86	*Pediococcus sp*	2.00	*Pediococcus acidilactici*
